# SSRD: Simple Sequence Repeats Database of the Human Genome

**DOI:** 10.1002/cfg.289

**Published:** 2003-06

**Authors:** Subbaya Subramanian, Vamsi M Madgula, Ranjan George, Satish Kumar, Madhusudhan W Pandit, Lalji Singh

**Affiliations:** 1 Centre for Cellular and Molecular Biology Uppal Road Hyderabad 500 007 India; 2 Ingenovis a Division of Ilabs Ltd 97 Road No. 3 Banjara Hills Hyderabad 500 034 India

## Abstract

Simple sequence repeats are predominantly found in most organisms. They play a major role in studies of genetic diversity, and are useful as diagnostic markers for many diseases. The simple sequence repeats database (SSRD) for the human genome was created for easy access to such repeats, for analysis, and to be used to understand their biological significance. The data includes the abundance and distribution of SSRs in the coding and non-coding regions of the genome, as well as their association with the UTRs of genes. The exact locations of repeats with respect to genomic regions (such as UTRs, exons, introns or intergenic regions) and their association with STS markers are also highlighted. The resource will facilitate repeat sequence analysis in the human genome and the understanding of the functional and evolutionary significance of simple sequence repeats. SSRD is available through two websites, http://www.ccmb.res.in/ssr and http://www.ingenovis.com/ssr.


**Comparative and Functional Genomics**
*Comp Funct Genom* 2003; **4**: 342–345.
Published online in Wiley InterScience (www.interscience.wiley.com). **DOI:** 10.1002/cfg.289

**Short Communication**

### SSRD: simple sequence repeats database of the human genome

Subbaya Subramanian[1], Vamsi M Madgula[2], Ranjan George[2], Satish Kumar[2], Madhusudhan W Pandit[1] and Lalji Singh[1]*

[1] *Centre for Cellular and Molecular Biology, Uppal Road, Hyderabad 500 007, India*
[2] *Ingenovis, a Division of Ilabs Ltd, 97 Road No. 3, Banjara Hills, Hyderabad, 500 034, India*

**Correspondence to:*
*Lalji Singh, Centre for Cellular and Molecular Biology, Uppal Road, Hyderabad 500 007, India.*
*E-mail: lalji@ccmb.res.in*

Received: 8 November 2003
Revised: 24 February 2003
Accepted: 28 February 2003

### Abstract

**Simple sequence repeats are predominantly found in most organisms. They play a major role in studies of genetic diversity, and are useful as diagnostic markers for many diseases. The simple sequence repeats database (SSRD) for the human genome was created for easy access to such repeats, for analysis, and to be used to understand their biological significance. The data includes the abundance and distribution of SSRs in the coding and non-coding regions of the genome, as well as their association with the UTRs of genes. The exact locations of repeats with respect to genomic regions (such as UTRs, exons, introns or intergenic regions) and their association with STS markers are also highlighted. The resource will facilitate repeat sequence analysis in the human genome and the understanding of the functional and evolutionary significance of simple sequence repeats. SSRD is available through two websites, http://www.ccmb.res.in/ssr and http://www.ingenovis.com/ssr. Copyright © 2003 John Wiley & Sons, Ltd.**

### Introduction

Among the different types of repeats, transposable elements are the most abundant and make up ca. 45% of the total human genome. The other major type of repeats is simple sequence repeats (SSRs), or microsatellites, which occupy about 3% of the human genome (International Human Genome Sequencing Consortium, 2001). SSRs are defined as regions within DNA sequences where short sequences (1–6 bp; monomers to hexamers) are repeated in tandem arrays. For example, a DNA stretch of GTGTGTGTGTGT would be referred to as $(GT)_6$. Alleles at a specific location (locus) can differ in the number of repeats. While the biological function of these SSRs is not known, many serve as a useful source of genetic markers. Microsatellites are found in coding and non-coding regions, play a significant role in genome evolution (Kashi *et al*., 1997), and may influence gene expression. One possible role of such non-coding DNA may be to regulate the expression of neighbouring genes by means of affecting chromatin organization. Strand slippage during replication has been suggested to be the most likely mechanism for the generation of mutations and polymorphisms in microsatellites (Pearson and Sinden, 1998). The other important aspect of these repeats is the instability of certain trinucleotide repeats that are known to cause neurodegenerative diseases and other genetic disorders (Sinden, 2001).

Taking into account the importance of SSRs, it becomes inevitable that there will be a need to analyse in detail the distribution of these repeats and genes associated with these repeats. Although there have been extensive studies on microsatellite repeats in humans (Dib *et al*., 1996; Wren *et al*., 2000) and various applications of SSRs such as studying genetic distance and constructing phylogenetic trees have been put forth (Slatkin, 1995), a complete inventory of the simple sequence repeats in the human genome is not available as a single resource. However, with the completion of the sequence of the human genome, this has

Copyright © 2003 John Wiley & Sons, Ltd.

become possible, and biologists involved in simple sequence repeat analysis could make extensive use of such a database.

### Methods

The complete human genome sequence, downloaded from the FTP site of Genbank (**ftp://ftp.ncbi.nlm.nih.gov/genomes/h_sapiens** — build number 29; 16 May 2002 release), was used to generate the simple sequence repeats data. The database contains the abundance, distribution and association of the 501 theoretically possible non-overlapping SSR types of k-mer repeats, where k ranges from one to six, i.e. monomer to hexamer (Jurka and Pethiyagoda, 1995). The number of 501 repeats was derived using the lowest alphabetical designations (i.e. for AC = CA = GT = TG, AC is given in the database). In this study, we have analysed the distribution of perfect simple sequence repeats (without indels) of length $\geq$12 base pairs (bp). This small cut-off value of 12 bp enables us to capture almost all of the SSRs present in the genome. A JAVA program, based on distributed computing and an exhaustive approach, was developed and used to scan the entire genome to determine the abundance and distribution of these repeats in coding and non-coding regions. The occurrences of repeats in genomic regions such as exons, introns and UTRs were identified based on the annotation of the human genome sequence in Genbank. Suitable links are provided with the NCBI and Genbank databases to the Accession and contig numbers that are provided in each table. The database will be updated when the new genome build becomes available.

### Organization of SSRD

SSRD can be accessed at **http://www.ccmb.res.in/ssr** and **http://www.ingenovis.com/ssr**. The complete data set is presented as the following views.

#### Repeat types across chromosomes

The data provided in this view gives the overall information on the repeat number and density for various repeat types ranging from monomer to hexamer. For simplicity, the repeats are grouped as per their 'k' mer value (e.g., monomer repeats A/T and G/C are grouped as one type). The table also provides statistics on the total occurrence for all repeat types across a particular chromosome, and for all chromosomes, for a particular repeat type.

#### 501 repeats across chromosomes

This view provides details on the occurrence and density of each of the 501 repeats across all human chromosomes. The density of each SSR is tabulated according to the repeat group. The user can select a chromosome and repeat group of interest to view the occurrence and density of a specific repeat type.

#### Repeat types across genomic regions

This view gives details on the distribution of SSRs across genomic regions (exons, introns and intergenic regions). First, the sizes of exons, introns and intergenic regions are calculated for each chromosome. Next, for each repeat found, the region to which it belongs is also captured. Thus, for a given chromosome, the density of repeat types and of specific repeats with respect to genomic regions is presented.

#### Details of each repeat

This view provides details of each SSR found in the human genome. For a given repeat, the repeat number and total length of the repeated sequence are given, along with the starting position of the repeat with respect to both the contig ID and the specific Accession ID. If the repeat is found in a particular gene, the name of the gene and the respective regions are displayed. In the event that a repeat lies in an intergenic region, the nearest downstream (sequence towards the 3′ region of the SSR) gene and the distance between the repeat and the gene is given. We have also presented the association of SSRs with sequence tagged sites (STSs) in terms of the distance between SSR and the known STS marker.

#### Details of repeats within genes

This view tabulates the genes that contain SSRs in their coding regions. This table is expected to give a comprehensive list of genes that may be associated with disease phenotypes, and will be helpful in the identification of novel genes that are involved in diseases related to repeat expansion.

Copyright © 2003 John Wiley & Sons, Ltd.

#### Details of each repeat for a specific gene

A search tool that fetches the details of microsatellite repeats for a specific gene is given in this view. The user can enter a gene name of interest to get the details of microsatellites that flank the gene, or repeats which are present within the gene. Repeats that are present both within the gene and in the flanking sequence of the gene are included in the search. In the case of repeats within the gene, the presence of SSRs in exons, introns and UTRs are tabulated. In the case of the SSRs that flank the gene, we have not specified any cut-off for the flanking sequence; instead we have included those SSRs in the flanking sequence on both sides of the gene until the end or the beginning of the next gene is encountered in the sequence. In this way, one can search for all of the SSRs that flank a particular gene.

### Discussion

SSRs are found in most genomes, with varying abundance (Toth *et al.*, 2000; Gur-Arie *et al.*, 2000). The analysis of various properties of SSRs will be helpful in understanding their biological significance. The density of each repeat type (length in base pairs contributed/Mb of genome) will reveal the abundance of each particular repeat type and the probable association of these repeat types with specific chromosomes. In our earlier studies we have found that the densities of SSRs across the human chromosomes are relatively uniform. However, the overall density of SSRs was found to be high on chromosome 19 (Subramanian *et al.*, 2003). Thangaraj *et al.* (2003) reported that the pentameric repeat GGAAT is predominantly associated with the human Y chromosome, in the Yq centromeric and heterochromatic regions, and may be involved in intrachromosomal recombination. Information on the abundance of SSRs, coupled with their distribution patterns in the coding as well as non-coding regions of the genome and their associations with genes and STS markers will shed more light on the function of SSRs in gene regulation. The location of the repeats with respect to contigs and accession numbers will serve as a map of these SSRs in the human genome, thereby associating them with particular disease phenotypes and developing newer molecular markers. As the STS markers are locus-specific, the distances (bp) provided in the database for those repeats which are appearing near such markers will be further helpful in mapping the location of the SSRs to their genomic locus.

The presence of microsatellites in the coding regions and regulatory regions of the genome can influence gene expression. The allelic variation of HUMTH01 (TCAT repeats) has been shown to be correlated with quantitative and qualitative changes in the binding of the ZNF191 protein, which contributes significantly to the control of the expression of quantitative genetic traits (Albanese *et al.*, 2001). Studies on GT tandem repeats on human chromosome 22 revealed that these repeats are associated with high recombination frequency (Majewski and Ott, 2000). One possible role of such non-coding DNA may be to regulate the expression of neighbouring genes by affecting chromatin organization. Hui *et al.* (2003) demonstrated that CA repeats in intron 13 of the human endothelial nitric oxide synthase (eNOS) gene function as an unusual intronic splicing enhancer, whose activity depends on the CA repeat number.

While there is no direct correlation between microsatellite content and genome size, it is generally believed that the microsatellite content of a genome depends on its size (Primmer *et al.*, 1997). Many markers have been developed from known sequences containing these repeats (available from databases and derived by screening genomic libraries). In spite of the recognition of simple repeats as markers and their association with gene regulation, the mechanisms underlying the allelic diversity of microsatellites are still poorly understood. Questions such as why certain repeat motifs are more common than others, and why there is variation of such repeats across taxa, are important from an evolutionary point of view.

### Acknowledgements

The authors would like to thank Sreedhar, Kavitha, Siva Prasad and Saritha for their support in developing the SSRD database. We are grateful to Rakesh Mishra and Ramesh Aggarwal for providing helpful discussions. We also thank the anonymous referees for their valuable suggestions. Financial support from CSIR and DBT is duly acknowledged.

Copyright © 2003 John Wiley & Sons, Ltd. 

### References

Albanese V, Biguet NF, Kiefer H, *et al*. 2001. Quantitative effects on gene silencing by allelic variation at a tetranucleotide microsatellite. *Hum Mol Genet* **10**: 1785–1792.

Dib C, Faure S, Fizames C, *et al*. 1996. A comprehensive genetic map of the human genome based on 5264 microsatellites. *Nature* **380**: 149–152.

Gur-Arie R, Cohen CJ, Eitan Y, *et al*. 2000. Simple sequence repeats in *Escherichia coli*: abundance, distribution, composition, and polymorphism. *Genome Res* **10**: 62–71.

Hui J, Stangl K, Lane WS, Bindereif A. 2003. HnRNP L stimulates splicing of the eNOS gene by binding to variable-length CA repeats. *Nature Struct Biol* **10**: 33–37.

Jurka J, Pethiyagoda C. 1995. Simple repetitive DNA sequences from primates: compilation and analysis. *J Mol Evol* **40**: 120–126.

International human genome sequencing consortium. 2001. Initial sequencing and analysis of the human genome. *Nature* **409**: 860–921.

Kashi Y, King D, Soller M. 1997. Simple sequence repeats as a source of quantitative genetic variation. *Trends Genet* **13**: 74–78.

Majewski J, Ott J. 2000. GT repeats are associated with recombination on human chromosome 22. *Genome Res* **10**: 1108–1114.

Pearson CE, Sinden RR. 1998. Trinucleotide repeat DNA structures: dynamic mutations from dynamic DNA. *Curr Opin Struct Biol* **8**: 321–330.

Primmer CR, Raudsepp T, Chowdhary BP, Moller AP, Ellegren H. 1997. Low frequency of microsatellites in the avian genome. *Genome Res* **7**: 471–482.

Sinden RR. 2001. Neurodegenerative diseases: Origins of instability. *Nature* **411**: 757–758.

Slatkin M. 1995. A measure of population subdivision based on microsatellite allele frequencies. *Genetics* **139**: 457–462.

Subramanian S, Mishra RK, Singh L. 2003. Genome-wide analysis of microsatellite repeats in human: their abundance and density in specific genomic regions. *Genome Biol* **4**: R13.

Thangaraj K, Subramanian S, Reddy AG, Singh L. 2003. Unique case of deletion and duplication in the long arm of the Y chromosome in an individual with ambiguous genitalia. *Am J Med Genet* **116**: 205–207.

Toth G, Gaspari Z, Jurka J. 2000. Microsatellites in different eukaryotic genomes: survey and analysis. *Genome Res* **10**: 967–981.

Wren JD, Forgacs E, Fondon JW III, *et al*. 2000. Repeat polymorphisms within gene regions: phenotypic and evolutionary implications. *Am J Hum Genet* **67**: 345–356.

Copyright © 2003 John Wiley & Sons, Ltd. 
